# Next-Generation Sequencing Workflow for NSCLC Critical Samples Using a Targeted Sequencing Approach by Ion Torrent PGM™ Platform

**DOI:** 10.3390/ijms161226129

**Published:** 2015-12-03

**Authors:** Irene Vanni, Simona Coco, Anna Truini, Marta Rusmini, Maria Giovanna Dal Bello, Angela Alama, Barbara Banelli, Marco Mora, Erika Rijavec, Giulia Barletta, Carlo Genova, Federica Biello, Claudia Maggioni, Francesco Grossi

**Affiliations:** 1 Lung Cancer Unit, IRCCS AOU San Martino-IST National Cancer Research Institute, L. go R. Benzi 10, 16132 Genoa, Italy; simona.coco@hsanmartino.it (S.C.); anna.truini@hsanmartino.it (A.T.); mariagiovanna.dalbello@hsanmartino.it (M.G.D.B.); angela.alama@hsanmartino.it (A.A.); ery80x@yahoo.it (E.R.); giulia.barletta@yahoo.it (G.B.); carlo.genova1985@gmail.com (C.G.); febiello@gmail.com (F.B.); claudia_87m@yahoo.it (C.M.); francesco.grossi@hsanmartino.it (F.G.); 2 Department of Internal Medicine and Medical Specialties (DIMI), University of Genoa, IRCCS AOU San Martino-IST National Cancer Research Institute, L. go R. Benzi 10, 16132 Genoa, Italy; 3 Laboratory of Molecular Genetics, IRCCS, Giannina Gaslini Institute, L. go G. Gaslini 5, 16148 Genoa, Italy; marta.rusmini@gmail.com; 4 Laboratory of Tumor Epigenetics, IRCCS AOU San Martino-IST National Cancer Research Institute, L. go R. Benzi 10, 16132 Genoa, Italy; barbara.banelli@hsanmartino.it; 5 Department of Pathology, IRCCS AOU San Martino-IST National Cancer Research Institute, L. go R. Benzi 10, 16132 Genoa, Italy; marco.mora@hsanmartino.it

**Keywords:** next-generation sequencing, NGS workflow, NSCLC, Ion Torrent PGM, FFPE, cfDNA

## Abstract

Next-generation sequencing (NGS) is a cost-effective technology capable of screening several genes simultaneously; however, its application in a clinical context requires an established workflow to acquire reliable sequencing results. Here, we report an optimized NGS workflow analyzing 22 lung cancer-related genes to sequence critical samples such as DNA from formalin-fixed paraffin-embedded (FFPE) blocks and circulating free DNA (cfDNA). Snap frozen and matched FFPE gDNA from 12 non-small cell lung cancer (NSCLC) patients, whose gDNA fragmentation status was previously evaluated using a multiplex PCR-based quality control, were successfully sequenced with Ion Torrent PGM™. The robust bioinformatic pipeline allowed us to correctly call both Single Nucleotide Variants (SNVs) and indels with a detection limit of 5%, achieving 100% specificity and 96% sensitivity. This workflow was also validated in 13 FFPE NSCLC biopsies. Furthermore, a specific protocol for low input gDNA capable of producing good sequencing data with high coverage, high uniformity, and a low error rate was also optimized. In conclusion, we demonstrate the feasibility of obtaining gDNA from FFPE samples suitable for NGS by performing appropriate quality controls. The optimized workflow, capable of screening low input gDNA, highlights NGS as a potential tool in the detection, disease monitoring, and treatment of NSCLC.

## 1. Introduction

Non-small cell lung cancer (NSCLC) is the leading cause of cancer-related death worldwide, and the majority of patients are diagnosed at advanced stages of NSCLC, with a dramatically poor five-year overall survival rate of 15% [1].

Recently, the identification of the molecular pathology of individual patient tumors has emerged as a promising opportunity for treatment of patients affected by advanced disease. Indeed, specific genetic alterations that drive cancer development can be targeted by selected inhibitors. In particular, the signaling pathway downstream of the Epidermal Growth factor Receptor (EGFR) has been investigated in advanced NSCLC patients harboring activating EGFR mutations (approximately 15% of Caucasian and 50% of Asian patients) treated with first-line tyrosine kinase inhibitors (TKIs), such as gefitinib, erlotinib, and afatinib. These patients report significant improvement in response and progression-free survival compared with patients treated with traditional chemotherapy [2].

Additionally, the application of molecular-based targeted approaches directed to other driver mutations, such as *KRAS*, *BRAF*, and the PI3K/AKT/mTOR pathway, is currently under evaluation, and several trials have been designed to assess the pharmacological efficacies of these compounds, reporting encouraging results [3].

Currently, traditional tools such as Sanger Sequencing (SS), real-time PCR, and pyosequencing (PS) can screen mutations on few genes per run with a sensitivity of approximately 20%, 10%, and 1%–2%, respectively [4,5,6,7]. However, the increasing number of novel potential targets results in more time-consuming and expensive diagnostic tests [8]. The advent of NGS has revolutionized the approach to detect gene mutations but requires high-quality DNA. The majority of advanced NSCLC tumor samples are usually represented by FFPE biopsies that result in limited quantity and low-quality DNA, affecting NGS feasibility. Recently, the evaluation of mutations in circulating free DNA (cfDNA) in the plasma of patients affected by advanced NSCLC has emerged as a hypothetical alternative approach to detect gene mutations from a liquid biopsy [9]. However, the feasibility of performing NGS on critical samples remains challenging, and increased efforts should be made in this direction to offer the best individual therapeutic option.

Here we present an optimized workflow to perform deep sequencing using the Ion AmpliSeq Colon and Lung Cancer Panel *v.*1 (Life Technologies, Carlsbad, CA, USA), which analyzes hotspot and targeted regions of 22 genes frequently mutated in colon and lung cancer. Analyses were performed on DNA resulting from critical samples, such as FFPE biopsy blocks and cfDNA, including a quality control step to assess the DNA quality from these samples.

## 2. Results

### 2.1. Assessment of NGS (Next-Generation Sequencing) Detection Limit, Specificity, Sensitivity, and Positive Predictive Value

gDNAs obtained from FFPE sections of engineered cell lines with clinical relevant mutations in tyrosine kinase domain of *EGFR*, such as p.T790M and the p.Glu746_Ala750del, were used to assess the limit of detection (LOD) of our NGS workflow. Firstly, dilution series of p.Thr790Met *EGFR* mutated gDNA with wild-type EGFR gDNA were used to obtain mutant allele frequencies of 50%, 10%, 5%, and 1%, respectively. All dilutions underwent to library preparation and sequenced by Ion PGM, achieving an average coverage per amplicon >1200× and uniformity >96% across the samples. Notably, the p.Thr790Met *EGFR* mutation was detected up to the third dilution (5%) with allele frequencies of 49.6%, 10.7%, and 5.6%, respectively, whereas the call of p.Thr790Met mutation was missed in the lowest dilution (1%). Similarly, we also assessed the LOD of our assay to correctly call the indel, applying a serial dilution of the p.Glu746_Ala750del gDNA with EGFR wild type, confirming the previous LOD of 5% also for indel detection. To evaluate the specificity, sensitivity, and Positive Predictive Value (PPV), we also sequenced two NSCLC cell lines (NCI-H1650 and NCI-H1975) and the two reference standards with EGFR mutation at 50% of frequency. NGS data analysis revealed a specificity of 100% for the detection of SNVs and INDELs compared to the genotypes of NCI-H1650 and NCI-H1975 cell lines (genotype data assessed by Coco *et al.*, using NGS platform) and the two FFPE reference standards (genotype data assessed by Horizon using Droplet Digital PCR), whereas the sensitivity for detecting variants at the LOD of 5% was 96% (Confidence Interval (CI) 95% = 80.5, 99.3) with PPV of 100% (95% CI = 86.7, 100.0).

### 2.2. FFPE (Formalin-Fixed Paraffin-Embedded) Quality Control

gDNA from FFPE tissues reported very high purity (both 260/280 and 260/230 ratios > 1.7) and underwent multiplex PCR-based quality control assay, with degradation status ranging from 0.3 to 0.8 Average Yield Ratio (AYR). To identify an AYR cut-off suitable for library preparation, we chose three FFPE DNA samples with different AYR values (0.3, 0.6, and 0.8) and prepared the libraries starting from 10 and 20 ng of gDNA. As expected, all samples were successfully amplified when starting from 20 ng, whereas only two out of three were amplified when starting from 10 ng; indeed, the sample that did not show any amplification products was the most fragmented (0.3 AYR, corresponding to 70% degradation). Additionally, to set the gDNA degradation status cutoff, as a prerequisite for proceeding to a downstream workflow, we increased the fragmentation status of gDNA with the lowest AYR (0.3) by heat. In more detail, by adjusting the fragmentation time we obtained a gDNA test with AYR of 0.2 that was amplified starting from 20 ng as input DNA for the library construction. However, this sample did not display any amplification products, suggesting that 0.3 AYR was a reliable decision criterion to predict the NGS suitability of FFPE samples.

Then, to evaluate the degradation effect on sequencing output, all positive libraries were sequenced. Notably, the sample with 0.6 AYR, although successfully amplified starting from 10 and 20 ng of gDNA, displayed much higher coverage (2/90 amplicons < 500×) using 20 ng compared with 10 ng (6/90 amplicons < 500×). Therefore, we decided to use a DNA input of 20 ng for the library construction of all FFPE DNA samples.

### 2.3. FFPE and Snap Frozen (SF) NGS

Twelve FFPE (AYR range 0.3–0.8) and matched SF gDNAs were sequenced. The coverage and uniformity of each sample run are reported in Supplementary Table S1. The total number of reads was 6,993,993 and 6,538,054 for SF and FFPE samples, respectively. NGS analysis disclosed an average number of reads per amplicon of 6475.9 in SF samples compared with 6050.5 in FFPE samples. Despite this high coverage, seven of 12 FFPE samples revealed at least one amplicon (range: 1–5) with coverage less than 500×, whereas only one SF sample showed one amplicon with unsatisfactory coverage. More specifically, the FFPE sample ID12 with an AYR = 0.3, showed 19 amplicons with a coverage <500×, suggesting that low quality of gDNA could affect sequencing results. Notably, the aforementioned seven FFPE DNAs reported recurrent amplicons not fully covered: AKT1_1 (chr14: 105246446-105246583), FGFR3_2 (chr4: 1806082-1806187), NOTCH1_1 (content; chr9: 139399338-139399447), STK11_3 (chr19: 1220481-1220603), and TP53_2 (chr17: 7579351-7579485). Interestingly, these amplicons displayed high GC content (greater than 61%). Notably, the coverage uniformity also correlated with gDNA fragmentation status; more specifically, all SF samples displayed 100% coverage uniformity, except for one with 98.9%, whereas FFPE samples ranged from 73.3% to 100%, referring to 0.4 and 0.8 AYR samples, respectively.

The Variant Caller (VC) plugin reported a total of 102 genetic variants (100 SNVs and two indels) (Supplementary Table S2). Notably, all the *EGFR* and *KRAS* mutations previously detected by SS were confirmed in all NSCLC samples reporting identical results starting from SF and matched FFPE tissue, although with different allele frequencies due to intra-tumor heterogeneity (Table 1), whereas the other seven genes present in the colon-lung panel showed discordant results between FFPE and matched SF samples. In particular, 11 discordant nucleotide substitutions were identified: six genetic variants at a frequency less than 5% (1 in SF; 5 in FFPE), and five ranging from 5% to 20% (three in SF; two in FFPE) (Supplementary Table S3).

**Table 1:** *EGFR* and *KRAS* mutational status comparison between Sanger Sequencing and NGS (Next-Generation Sequencing) Variant Caller software. This table reports the *EGFR* or *KRAS* mutational status, previously defined by Sanger Sequencing from the 12 patients compared to the Variant Caller NGS results. The *EGFR* or *KRAS* mutation nomenclature is based on the convention recommended by the Human Genome Variation Society [10,11].

Patient	Tumor Type	*EGFR* and *KRAS* Status by Sanger Sequencing	*EGFR* and *KRAS* Status Confirmed by VC Software	Allele Name
1	SF	NM_033360.3 (KRAS): c.34G>C	Confirmed *KRAS* mutation	COSM518
		(p.Gly12Arg)	AF: 29.8%	
1	FFPE	NM_033360.3 (KRAS): c.34G>C	Confirmed *KRAS* mutation	COSM518
		(p.Gly12Arg)	AF: 58.6%	
2	SF	NM_033360.3 (KRAS): c.34G>T	Confirmed *KRAS* mutation	COSM516
		(p.Gly12Cys)	AF: 24.1%	
2	FFPE	NM_033360.3 (KRAS): c.34G>T	Confirmed *KRAS* mutation	COSM516
		(p.Gly12Cys)	AF: 6.4%	
3	SF	NM_005228.3 (EGFR): c.2239_2240TT>CC	Confirmed *EGFR* mutation	COSM24267
		(p.Lys747Pro)	AF: 37.8%	
3	FFPE	NM_005228.3 (EGFR): c.2239_2240TT>CC	Confirmed *EGFR* mutation	COSM24267
		(p.Lys747Pro)	AF: 45.6%	
4	SF	NM_033360.3 (KRAS): c.35G>T	Confirmed *KRAS* mutation	COSM520
		(p.Gly12Val)	AF: 24.0%	
4	FFPE	NM_033360.3 (KRAS): c.35G>T	Confirmed *KRAS* mutation	COSM520
		(p.Gly12Val)	AF: 23.2%	
5	SF	NM_005228.3 (EGFR): c.2237_2255delinsT	Confirmed *EGFR* mutation	COSM12384
		(p.Glu746_Ser752delinsVal)	AF: 5.6%	
5	FFPE	NM_005228.3 (EGFR): c.2237_2255delinsT	Confirmed *EGFR* mutation	COSM12384
		(p.Glu746_Ser752delinsVal)	AF: 10.5%	
6	SF	NM_005228.3 (EGFR): c.2236_2250del	Confirmed *EGFR* mutation	COSM6225
		(p.Glu746_Ala750del)	AF: 7.8%	
6	FFPE	NM_005228.3 (EGFR): c.2236_2250del	Confirmed *EGFR* mutation	COSM6225
		(p.Glu746_Ala750del)	AF: 45.8%	
7	SF	NM_033360.3 (KRAS): c.35G>A	Confirmed *KRAS* mutation	COSM521
		(p.Gly12Asp)	AF: 38.6%	
7	FFPE	NM_033360.3 (KRAS): c.35G>A	Confirmed *KRAS* mutation	COSM521
		(p.Gly12Asp)	AF: 26.3%	
8	SF	NM_033360.3 (KRAS): c.34G>T	Confirmed *KRAS* mutation	COSM516
		(p.Gly12Cys)	AF: 50.5%	
8	FFPE	NM_033360.3 (KRAS): c.34G>T	Confirmed *KRAS* mutation	COSM516
		(p.Gly12Cys)	AF: 24.5%	
9	SF	NM_033360.3 (KRAS): c.34G>T	Confirmed *KRAS* mutation	COSM516
		(p.Gly12Cys)	AF: 42.0%	
9	FFPE	NM_033360.3 (KRAS): c.34G>T	Confirmed *KRAS* mutation	COSM516
		(p.Gly12Cys)	AF: 41.7%	
10	SF	NM_005228.3 (EGFR): c.2573T>G	Confirmed *EGFR* mutation	COSM6224
		(p.Leu858Arg)	AF: 45.3%	
10	FFPE	NM_005228.3 (EGFR): c.2573T>G	Confirmed *EGFR* mutation	COSM6224
		(p.Leu858Arg)	AF: 40.2%	
11	SF	NM_033360.3 (KRAS): c.37G>T	Confirmed *KRAS* mutation	COSM527
		(p.Gly13Cys)	AF: 32.0%	
11	FFPE	NM_033360.3 (KRAS): c.37G>T	Confirmed *KRAS* mutation	COSM527
		(p.Gly13Cys)	AF: 24.2%	
12	SF	NM_033360.3 (KRAS): c.34G>T	Confirmed *KRAS* mutation	COSM516
		(p.Gly12Cys)	AF: 31.0%	
12	FFPE	NM_033360.3 (KRAS): c.34G>T	Confirmed *KRAS* mutation	COSM516
		(p.Gly12Cys)	AF: 42.6%	

Abbreviations: SF: Snap Frozen; FFPE: Formalin-Fixed Paraffin-Embedded; VC: Variant Caller; AF: Allele Frequency.

To confirm the Single Nucleotide Variants (SNVs) (excluding indels) identified by the VC software, we performed a second round of analysis using the GATK software, which identified 103 nucleotide substitutions. Notably, GATK validated 93 SNVs identified by VC: 89 FFPE-SF matching SNVs and four of five VC-SNVs (5%–20% frequency) FFPE-SF non-matching, reported as pathogenic mutations (COSM516; COSM1579024; COSM710; COSM11496). Conversely, this pipeline did not confirm the six VC-SNVs (<5% frequency) and one VC-SNV (5%–20% frequency). Moreover, the GATK software identified an additional 10 non-matching novel SNVs (six with a frequency <20% and four with a frequency higher than 20%; range: 20%–35%). However, since none of those four (>20%) was confirmed by SS, we consider them as false positives (data not shown).

We also applied the PS to further validate the five genetic variants (5%–20% frequency) discordant between the FF and matching FFPE samples, confirming all VC-SNVs as true positives (Supplementary Table S3). Overall these data suggest the superiority of the VC pipeline to the call genetic variants generated by Ion PGM. Therefore, we considered 94 SNVs and two indels called by VC to define the tumor samples’ mutational profile, excluding six genetic variants at a frequency lower than 5%, according to our assay LOD.

**Figure 1:**
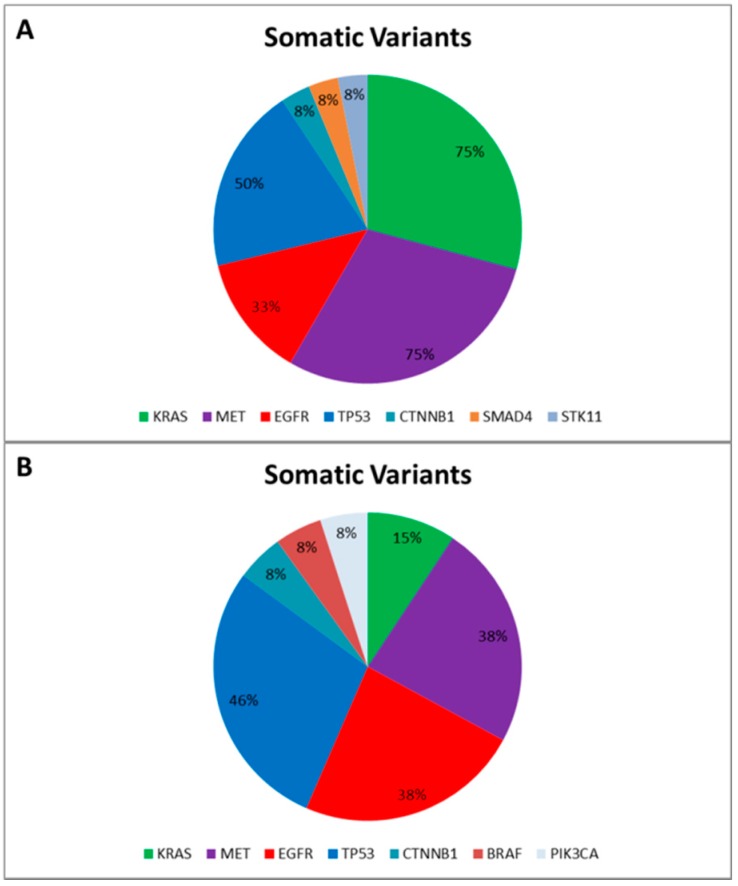
Somatic Variants identified in the “Training Set” (**A**) and in the “Validation Set” (**B**), respectively. The pie charts report the frequency of mutated genes in the “Training Set” (**A**) and in the “Validation Set” (**B**) based on the Catalogue of Somatic Mutation in Cancer (COSMIC) [13].

Data reported an average of 7.4 genetic variants (range 5–12) per patient, including Single Nucleotide Polymorphisms (SNPs) and pathogenic mutations. More specifically, only two SNPs involving the *DDR2* intron region (rs200983597 and rs143729297) reported a Minor Allele Frequency (MAF) smaller than 0.01 defined as “rare” variants [12]. Among the pathogenic variants (Figure 1A), tumor protein p53 (*TP53*) was the most altered gene: five out of 12 patients harbored pathogenic mutations (COSM44571; COSM43814; COSM45413; COSM40942; COSM10769) and all reported a SNP (rs1042522). Additionally, five patients harbored *MET* proto-oncogene receptor tyrosine kinase (*MET*) gene mutations (COSM706; COSM710; COSM1579024). Notably, COSM710 and COSM1579024 coexisted with *KRAS* mutations (COSM518; COSM520; COSM516). Further somatic mutations involved the serine/threonine kinase 11 (COSM49004), catenin (cadherin-associated protein) beta 1 (COSM5670), and SMAD family member 4 (COSM14151).

Finally, to correctly call genetic variants also including indel, we sequenced a further cohort of 13 NSCLC patients, previously tested for *EGFR* mutational status by SS in order to validate our optimized workflow and test the ability of the bioinformatic pipeline. All gDNA underwent to a multiplex-PCR quality control step, reporting AYR values greater than 0.3, predictive of NGS suitability. Then all FFPE gDNAs were subjected to the Ion PGM workflow and successfully sequenced. In particular, the data analysis properly called four *EGFR* variants detected by SS. Similarly to the training set, the data analysis identified a mean of 7.0 genetic variants (ranging from five to 10) per patient, and, besides the *EGFR* activating mutations, we identified further pathogenic variants involving *BRAF*, *CTNNB1*, *KRAS*, *MET*, *PI3KCA*, and *TP53* genes (Figure 1B).

### 2.4. cfDNA NGS

Plasma samples, collected at diagnosis, were available for nine out of 12 patients for cfDNA extraction. Quantification of cfDNA was evaluated by qPCR using the *hTERT* assay, showing total concentrations ranging from 0.08 to 10 ng. Because the gDNA input recommended for library construction is 10 ng, we used a Whole Genome Amplification (WGA) technique to increase the starting cfDNA amount. First, we evaluated the WGA technique performance by a scalar dilution of NCI-H1650 human cell line gDNA (10, 1, and 0.1 ng), reporting final gDNA yields of approximately 20 μg regardless of the starting gDNA input. Then, 10 ng of each WGA NCI-H1650 was sequenced to evaluate the specificity and sensitivity of the WGA technique. The sequencing outputs reported a coverage of WGA gDNA greater than 500× and uniformity of 95.2%, except for the WGA 0.1 ng input gDNA sample that displayed four unsatisfactory covered amplicons and a uniformity of 81.1%. The NGS data analysis showed an optimum variant call concordance and identical results in terms of frequencies of variant calls starting from 10, 1, and 0.1 ng of NCI-H1650 gDNA (data not shown). However, the comparison between WGA and the original unamplified NCI-H1650 gDNA displayed five novel insertions in common among all WGA samples. Because these insertions occurred in homopolymeric regions (five or more nucleotide repeats), we hypothesized them to be amplification artefacts due to the WGA technique. Additionally, four out of five novel insertions displayed an AF lower than our LOD (5%); therefore, these insertions were excluded from the performance analysis of NGS workflow on WGA samples resulting in specificity, sensitivity, and PPV of 87.5% (CI 95% = 52.9, 97.7), 100.0% (CI 95% = 67.5, 100), and 100.0% (CI 95% = 67.5, 100), respectively. Therefore, due to the introduction of amplification artefacts in WGA cell lines, we decided to limit the use of the WGA approach to the three samples characterized by low input cfDNA (<1 ng). The remaining six samples were then enriched starting from a lower amount of recommended input cfDNA (2–10 ng) by increasing the cycle number of library PCRs. This modified protocol allowed us to obtain suitable no-WGA libraries; the coverage and uniformity of each run are displayed in Supplementary Table S1.

Although the average reads per amplicon for WGA cfDNA was higher compared with no-WGA cfDNA samples (6719.7 *vs.* 4858.5), the coverage uniformity was better in the no-WGA cfDNA; more specifically, WGA cfDNA showed an average of 83.7% (81.1%–85.6%) *vs.* 99.4% of the no-WGA (98.9%–100%). Indeed, no-WGA cfDNA sample exhibited coverage greater than 500× for all amplicons, except for one sample with 64 amplicons <500×, likely because of a low input of cfDNA (2.1 ng). Conversely, WGA cfDNAs always displayed five amplicons with coverage less than 500×. Notably, as previously observed in the seven FFPE samples, the recurrent uncovered amplicons (AKT1_1, FGFR3_2, NOTCH1_1, STK11_3, and STK11_5) contained high GC-content. Data obtained from VC were compared to the matched tumor mutational profile. As expected, the WGA cfDNA displayed additional insertions; notably, these insertions, likely due to non-specific amplification artefacts of WGA, were the same observed in the NGS analysis of WGA NCI-H1650 and therefore filtered out. Moreover, the comparison analysis identified two additional SNVs with frequencies of 4.4% and 20.6%. However, the second variant call by GATK (Supplementary Table S4) validated all SNVs identified by VC, excluding the nucleotide variant below 5%. Although the remaining discordant variant (20.6%) was confirmed by GATK, it occurred in a WGA-cfDNA sample, suggesting a possible PCR artefact. Unexpectedly, eight out of nine cfDNAs did not disclose any somatic mutations either in the gatekeeper gene *TP53* or in the other genes mutated in their matched tumors; by contrast, one cfDNA from a patient with metastatic disease presented the identical mutational profile reported in the corresponding tumor.

## 3. Discussion

**Figure 2:**
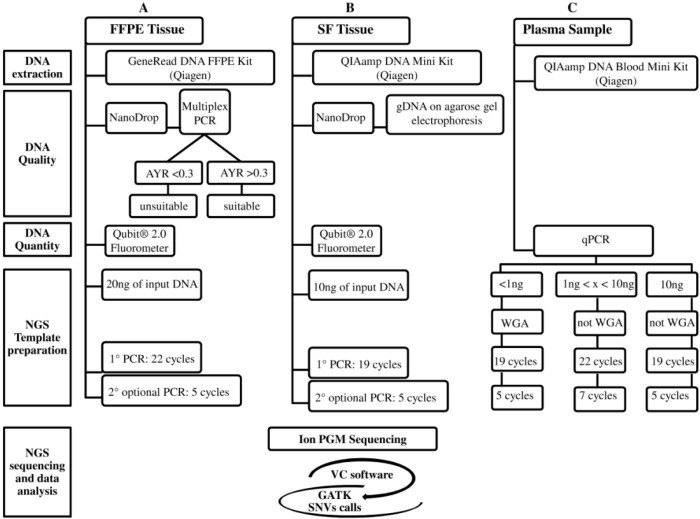
The three analytical NGS (Next-Generation Sequencing) workflows are: (**A**) FFPE (Formalin-Fixed Paraffin-Embedded); (**B**) SF (Snap Frozen); and (**C**) Plasma. These workflows, currently designed for the Ion PGM platform, are divided into five steps: DNA extraction, gDNA Quality, gDNA Quantity, NGS Template preparation, and NGS sequencing and data analysis. The latter, in common among three workflows, includes: Ion PGM sequencing and variant identification using VC software and then GATK for SNVs call. The NGS cost (from DNA extraction to NGS data analysis), excluding personnel, is approximately 250€ for each FFPE, SF, and plasma sample (**A**–**C**). The NGS turnaround time for processing SF (**B**) and no-WGA cfDNA (**C**) samples is approximately five days, whereas for FFPE (**A**) and WGA cfDNA (**C**) samples one more day is required for the multiplex PCR DNA quality control (**A**) and WGA technique (**C**).

Recently, targeted therapy has emerged as a promising therapeutic option for the subgroup of advanced NSCLC patients carrying activating mutations in druggable genes [2]. Currently, the screening of clinically-actionable mutations is performed on FFPE tumor biopsies, but the amount of tumor tissue is often limited, and the gDNA quality may not be always optimal. Moreover, the increasing number of candidate targets requires the validation of highly sensitive throughput technologies, such as NGS, into clinical settings. To date, several studies have investigated targeted deep sequencing on FFPE samples from different cancer types [14,15,16], including NSCLC [17]; here, we propose an optimized workflow using a specific 22-gene enrichment lung cancer panel to successfully sequence critical samples, such as FFPE biopsies and cfDNA, assuring good quality of NGS data. The workflow includes correct quantification and quality control of input gDNA, a WGA approach to linearly increase the gDNA for low input of starting gDNA (<1 ng), library protocols tailored for specific gDNA type (FFPE and cfDNA), and a robust bioinformatic pipeline to correctly call both SNVs and indels (Figure 2). Similarly to other NGS workflows [18], our assay was able to detect up to 5% of AF either SNV and indel, achieving an optimal assay performance for FFPE samples (100% of specificity and PPV; 96% of sensitivity). The NGS workflow was retrospectively evaluated on DNA extracted from SF and matching FFPE and cfDNA belonging to 12 resected NSCLC patients previously tested for *EGFR* and *KRAS* mutations by SS.

Pre-analytic quality control to define suitable DNA for NGS application is crucial, particularly when it is applied to highly degraded DNA, such as FFPE DNA. The majority of studies, aimed to define FFPE deep sequencing guidelines, focused on the correct DNA quantification method [19,20], whereas our workflow, which involves a multiplex-PCR approach, defined an AYR parameter, indicative of gDNA degradation status, that highly correlates with sequencing quality metrics, such as uniformity and coverage. Notably, we identified an integrity threshold for the processing of FFPE gDNAs estimated at 0.3 AYR, which corresponds to 70% of gDNA fragmentation. Moreover, although the library protocol requires 10 ng of input FFPE gDNA, we demonstrated that when doubling the input FFPE DNA amount (20 ng), highly fragmented DNAs up to 0.3 AYR were successfully sequenced. Notably, this improvement increased the number of amplicons covered to at least 500×, a parameter required for any clinically-oriented cancer panel [18]. However, we identified five recurrent amplicons that were insufficiently covered in highly-degraded FFPE gDNAs because of high GC content (>60%), as previously reported [21].

One critical point of the NGS analysis is generating a list of trustworthy variants; therefore, we tested whether the integration of two different bioinformatic pipelines could improve the SNVs call with higher sensitivity and accuracy compared with single software performance. Specifically, we compared the VC, the Ion PGM plugin with optimized pre-set parameters, with GATK, one of the most used free NGS tools. Each pipeline showed different results in the detection of SNVs (100 SNVs by VC *vs.* 103 by GATK (95 in common between the two pipelines). Differences between the pipelines could be due to the different alignment and calling algorithms [22]. Furthermore, the GATK software exhibited a very high error rate in indel calling (approximately 20 indels per sample; data not shown), leading us to exclude it in the indel calling. A possible explanation is that the GATK pipeline was developed for Illumina data analysis, in contrast to the VC pipeline that has been set up for the analysis of FastQ data generated from Ion-platforms [23]. In this context, some evidence has been previously reported regarding the high rate of false positive variants detected by GATK with non Illumina-FastQ data. These false positives particularly refer to small insertions and deletions (indels) in homopolymeric regions where the difficulty in detecting and determining the genotype of short indels remains a challenge because of the propensity for polymerase slipping during PCR, resulting in sequence artifacts that GATK cannot distinguish [24]. The validation with the PS confirmed all VC discordant genetic variants between SF and matching FFPE samples (>5% frequency), demonstrating that the VC pipeline is optimized for Ion PGM data. Indeed, these discordant variants among SF and matching FFPE samples may be explained by intra-tumor heterogeneity, as previously observed in NSCLC [25,26,27,28,29]. Although the integration of GATK did not exclude any false positive SNVsby VC, it removed all the SF/FFPE discordant SNVs detected by the VC pipeline with frequencies less than 5%, confirming our LOD assay.

*EGFR* and *KRAS* somatic mutations, previously detected by SS, were confirmed by both pipelines either in SF and matched FFPE, validating the high specificity of the Ion AmpliSeq Colon and Lung Cancer panel for the identification of these mutations. Our data revealed that *TP53* was the most frequent pathogenically-mutated gene [30,31], and all patients harbored the SNP rs1042522 in *TP53*. Interestingly, this SNP, responsible for a substitution of a proline residue with an arginine residue in codon 72 of *TP53*, has been associated with lung cancer susceptibility [32] and a risk of toxicity during platinum-based chemotherapy treatment in advanced NSCLC patients [33]. Notably, we also observed a recurrent association between *MET* genetic variants and *KRAS* activating mutations, as already described in NSCLC by Govindan *et al.* [34]. Due to its high sensitivity, NGS represents an optimal approach to investigate the mutational profile of cfDNA as a non-invasive diagnostic approach to monitor disease progression during treatment. Indeed, a fraction of cfDNA derives from tumor cells and is referred to as ctDNA in cancer patients [35]. To date, several studies have demonstrated the feasibility of using ctDNA as a potential tumor marker in a limited number of patients with various solid cancers; however, few studies focused on NSCLC have been performed [9]. Therefore, we developed an optimized workflow for cfDNA mutational screening, combining a WGA approach for low input samples (<1 ng) and a specific protocol for sequencing library construction, resulting in high numbers of good-quality reads. Currently, several WGA approaches, differing in assay chemistry and complexity, have been developed [36,37,38,39,40]. Here, we used a WGA based on the MDA technology that employs the phi29 DNA polymerase and random primers to amplify gDNA without thermocycling [40,41]. Several studies revealed that MDA can amplify the genome with higher uniformity, longer length, and less bias compared with PCR-based WGA methods [41,42,43,44,45]. Therefore, MDA-based WGA has been shown to be the best method to obtain high quality cfDNA for different applications, such as the NGS [44,46,47]. Another important concern of the WGA approach is the correct quantification of the amplified DNA. Indeed, WGA techniques report non-specific side products and residual WGA reagents that may influence DNA quantification by fluorometric methods [43,48]. Therefore, we suggested performing WGA-DNA quantification using real-time PCR, which has been demonstrated to be the most reliable method [49]. The sequencing output obtained from the WGA NCI-H1650 displayed a similar performance to the standard no-WGA cell line, irrespective of input gDNA. Similar SNV frequencies were reported across the WGA NCI-H1650 samples, suggesting that this technology may be applied to the colon and lung assay when the starting gDNA amount is limited. However, it should be taken into account that the MDA-based WGA introduces some amplification biases that mainly occur in DNA homopolymeric regions, as previously observed, resulting in a lower specificity of the assay [41,50,51]. Because the WGA application might be necessary to yield sufficient DNA for the subsequent sequencing workflow [52], we tried to limit all critical points. Our optimized workflow and NGS analysis on cfDNAs allowed for detection of the same mutational profile of the corresponding tumor in one cfDNA sample. However, among nine NSCLC patients with available plasma, only two were at stage IV of disease. Because several studies revealed that the presence of ctDNA was mainly detected in aggressive disease [9,53], this observation could explain why we detected ctDNA in only one of two patients with advanced NSCLC. The unidentified somatic mutations in localized (stage I–III) cfDNA samples could also be explained by the detection limit of mutation allele frequencies of our NGS assay, estimated at 5% [54].

However, since the Ion PGM technology utilizes scalable semiconductor chips, a lower number of samples in the same run could increase the amplicon coverage; this might allow for the detection of variants with lower frequency (<5%) in the hypothesis to also apply the NGS workflow to the ctDNA screening. Such higher throughput, might also permit us to detect tumor heterogeneity e.g., in the profiling of ctDNA and to call variants at very low frequencies. Furthermore, since the correct amplicon size is critical to the success of sequencing experiments, designing custom panels with a shorter length of amplicons could improve the sequencing performances, especially for highly fragmented DNA such as FFPE and cfDNA. Finally, it should be kept in mind that customizing panels for a subset of druggable genes of a specific cancer type may offer testing at a lower cost, thereby yielding a higher coverage.

## 4. Experimental Section

### 4.1. Sample Collection

Twelve patients who underwent surgery for NSCLC at different stage of the disease were enrolled in our study as the “Training Set”, based on their previously known *EGFR* and *KRAS* mutational statuses. The clinical characteristics of the patients are reported in Table 2. SF tumors from all patients were available in the tissue biobank of our Institute. Additionally, matched FFPE tissues were obtained from the pathology archive. All samples were evaluated by a pathologist for malignant cell content to macrodissect and enrich the tumor component up to 50%, when necessary. Nine of the 12 plasma samples from patients were also collected at time of diagnosis.

We also enrolled a further 13 NSCLC patients as the “Validation Set” (Table 3), and for each case the FFPE tissue block was revised by the pathologist to verify the tumor cell content. Moreover, three Horizon FFPE standards (*EGFR* p.Thr790Met, *EGFR* p.Glu746_Ala750del, and *EGFR* wild-type reference standards; Horizon, Cambridge, UK) and two human NSCLC cell lines (NCI-H1650 p.Glu746_Ala750del and NCI-H1975 p.Thr790Met/p.Leu858Arg; American Type Culture Collection, Manassas, VA, USA) were used to assess the parameter performances of our assay. The present study was approved by the local ethics committee (TP-01-2014; 255REG2014) and written informed consent was obtained from every patient.

**Table 2:** Clinical characteristics of NSCLC (Non-small cell lung cancer) in the “Training Set”.

Patient	Block Age	% TC	SF	FFPE	cfDNA	Histology	TNM	Stage	Age at Diagnosis	G	Smoking Habits	PS	OS	Status
1	2012	>50	x	x	x	SCC	T1b, N0, M0	IA	77	M	S	1	30	A
2	2010	>50	x	x	x	ADK	T1a,N2,M0	IIIA	67	M	NS	1	18	DOD
3	2013	>50	x	x	x	ADK	T2, N2, M1a	IV	76	F	NS	1	14	DOD
4	2010	>50	x	x	x	ADK	T2b,N0,MX	IIA	76	M	FS	1	11	DOD
5	2013	>50	x	x	NA	ADK	T2a,N2,M1b	IV	75	F	NS	1	13	A
6	2011	>50	x	x	NA	ADK	T2a, N0, MX	IB	78	F	NS	1	29	DOD
7	2013	>50	x	x	x	ADK	T2a, N2, M1b	IV	65	M	S	1	3	DOD
8	2013	>50	x	x	x	ADK	T2b, N1, M0	IIB	59	F	S	1	23	A
9	2012	>50	x	x	x	ADK	T2a, N0, MX	IB	72	M	FS	0	28	A
10	2011	>50	x	x	NA	ADK	T2b, N0, M0	IIB	66	F	FS	1	42	A
11	2010	>50	x	x	x	ADK	T1a, N0, M0	IA	77	M	FS	0	49	DOD
12	2010	>50	x	x	x	ADK	T1a, N0, M0	IA	60	F	S	1	32	DOD

**Table 3:** Clinical characteristics of NSCLC patients in the “Validation Set”.

Patient	Block Age	% TC	SF	FFPE	cfDNA	Histology	TNM	Stage	Age at Diagnosis	G	Smoking Habits	PS	OS	Status
13	2015	>50	NA	x	NA	ADK	T2, N2, M0	IIIB	59	M	S	0	5	A
14	2014	>50	NA	x	NA	ADK	Tx, Nx, M1a	IV	42	M	NS	0	14	A
15	2015	>50	NA	x	NA	ADK	T3, N3, M1a	IV	64	M	FS	1	4	A
16	2014	>50	NA	x	NA	ADK	T4, N2, M1b	IV	77	F	NS	0	12	A
17	2014	>50	NA	x	NA	ADK	T3, N2, M0	IIIA	70	F	NS	1	8	A
18	2012	>50	NA	x	NA	ADK	T3, N2, M1b	IV	63	M	S	1	37	A
19	2014	>50	NA	x	NA	ADK	T4, N2, M1b	IV	70	M	FS	1	2	DOD
20	2014	>50	NA	x	NA	ADK	T4, N0, M0	IIIA	68	M	FS	0	16	A
21	2011	>50	NA	x	NA	ADK	T1a, N0, M0	IA	71	F	NS	1	42	DOD
22	2014	>50	NA	x	NA	ADK	T2, N2, M1b	IV	81	F	NS	1	10	DOD
23	2013	>50	NA	x	NA	ADK	T1b, N2, M0	IIIA	72	M	FS	1	22	A
24	2014	>50	NA	x	NA	ADK	T3, N3, M1b	IV	61	M	NS	1	11	DOD
25	2015	>50	NA	x	NA	ADK	Tx, Nx, M1b	IV	55	F	FS	0	7	A

Abbreviations: TC: Tumor Content; ADK: Adenocarcinoma; SCC: squamous cell carcinoma; TNM: Tumor-Nodes-Metastasis; G: Gender; F: Female; M: Male; S: Smoker; NS: never smoker; FS: Former smoker; PS: Performance Status; OS: Overall Survival (Month); A: Alive; DOD: Dead of disease; x: present; NA: not available.

### 4.2. DNA Extraction and Quality Control

gDNA from SF samples and the NCI-H1650 cell line was isolated using a QIAamp^®^ DNA Mini Kit (Qiagen, Hilden, Germany), whereas gDNA from FFPE tissues was extracted using the GeneRead DNA FFPE Kit (Qiagen), according to the manufacturer’s recommendations. All gDNA samples were then quantified by a Qubit^®^ 2.0 Fluorometer (Invitrogen, Carlsbad, CA, USA) using a Qubit^®^ dsDNA HS Assay Kit. cfDNA was extracted from 400 µL of plasma with a QIAamp DNA Blood Mini Kit (Qiagen) and quantified by quantitative PCR (qPCR) using the human telomerase reverse transcriptase (*hTERT*) TaqMan^®^ Copy Number Reference Assay (Catalog: 4403316; Life Technologies). The calibration curve was defined based on a dilution series (10–1 × 10^6^ pg) of a control human genomic DNA standard (Promega, Madison, WI, USA). The qPCR reaction was performed in a final reaction volume of 10 µL using 5 µL of TaqMan Universal Mastermix (Life Technologies, Carlsbad, CA, USA), 1 µL of the TaqMan^®^ Copy Number Reference Assay *hTERT* (Life Technologies), and 4 µL of cfDNA on a RealPlex (Eppendorf, Hamburg, Germany) device. Each sample was run in duplicate, and the final concentration calculated by interpolation of the CT value with the aforementioned calibration curve. Both SF and FFPE gDNA underwent a quality control assay. DNA quality was first assessed using a NanoDrop ND-1000 (Thermo Scientific, Wilmington, DE, USA) to measure the whole absorption spectrum (220–750 nm) and calculating absorbance ratios at both 260/280 and 260/230. SF gDNA was analyzed on 0.8% agarose gel electrophoresis to visualize the DNA size distribution, whereas we performed a multiplex PCR-based quality control assay using a slight modification of the van Beers *et al.* [55] protocol to assess FFPE gDNA fragmentation status. Briefly, 30 ng of FFPE gDNA, as measured by Qubit, was analyzed by a multiplex PCR reaction performed using three independent sets of *GAPDH* primers that amplify 200, 300, and 400 bp fragments. The amplification was performed in a reaction volume of 30 µL with final concentrations of 0.133 µM of each primer, 1.5 U AmpliTaq Gold^®^ DNA Polymerase (Life Technologies, Carlsbad, CA, USA), GeneAmp^®^ 10× PCR Gold Buffer, 1.5 mmol/L MgCl_2_, and 0.2 mmol/L dNTPs. PCR was conducted in a Mastercycler^®^ nexus Thermal Cycler (Eppendorf) as follows: 95 °C for 7 min, 35 cycles of 1 min at 94 °C, 1 min at 56 °C, and 3 min at 72 °C, followed by a hold at 72 °C for 7 min. PCR products were then analyzed with an Agilent 2100 Bioanalyzer using the Agilent High Sensitivity DNA Kit (Agilent Technologies, Santa Clara, CA, USA). The yield of the resulting three amplicons was evaluated by comparison with the yield of amplicons from a reference DNA template (Promega). The Average Yield Ratio (AYR), calculated for each amplicon, was used as a quantitative indicator of FFPE DNA integrity.

### 4.3. Whole Genome Amplification (WGA)

cfDNA less than 1 ng, estimated by qPCR, underwent WGA using the REPLI-g Single Cell Kit (Qiagen). The REPLI-g Single Cell Kit is based on a Multiple Displacement Amplification (MDA) technology that achieves highly uniform amplification across the entire genome with minimal locus bias and reduced nonspecific amplification artefacts [41]. To evaluate the performance of this assay for subsequent NGS application, we previously amplified a scalar dilution of NCI-H1650 cell line gDNA (10, 1, and 0.1 ng). The amplified gDNAs were diluted 1:100 and evaluated by qPCR using the *hTERT* assay, as previously described.

### 4.4. Next Generation Sequencing (NGS)

Libraries were amplified using the Ion AmpliSeq Colon and Lung Cancer Panel *v.*1 (Life Technologies), which analyzes 90 amplicons in hotspots and target regions of 22 genes (*AKT1*, *ALK*, *BRAF*, *CTNNB1*, *DDR2*, *EGFR*, *ERBB2*, *ERBB4*, *FBXW7*, *FGFR1*, *FGFR2*, *FGFR3*, *KRAS*, *MAP2K1*, *MET*, *NOTCH1*, *NRAS*, *PIK3CA*, *PTEN*, *SMAD4*, *STK11*, and *TP53*) with a coverage of over 500 mutations involved in colon and lung cancers. Overall, 20 ng for FFPE, 10 ng for SF gDNA and WGA cfDNA, and a range from 1 to 10 ng for no-WGA cfDNA were amplified using the Ion AmpliSeq™ Library Kit 2.0 (Life Technologies), barcoding each sample. Different cycling conditions were performed according to the DNA type: 22 cycles for FFPE gDNA and no-WGA cfDNA and 19 cycles for SF gDNA and WGA cfDNA in the first multiplex PCR, whereas in the second, optional PCR, the SF, FFPE and WGA cfDNA were subjected to five cycles, and the no-WGA cfDNA underwent seven cycles.

The library size was checked using the Agilent High Sensitivity DNA Kit by the Bioanalyzer 2100 instrument (Agilent Technologies), and library concentration was evaluated with a Qubit^®^ 2.0 Fluorometer using the Agilent High Sensitivity DNA Kit (Life Technologies). Each diluted library (100 pM) was amplified through emulsion PCR using the OneTouch™ Instrument (Life Technologies) and enriched by the OneTouch™ ES Instrument (Life Technologies) using the Ion PGM Template OT2 200 KIT, following the manufacturer’s instructions. Finally, sequencing was performed on the Ion PGM (Life Technologies) with the Ion PGM 200 Sequencing Kit (Life Technologies), loading barcoded samples into a 314*v.*2 or 316*v.*2 chip.

### 4.5. NGS Data Analysis

The PGM sequencing data were analyzed by the Ion Torrent Software Suite *v.*4.2 (Life Technologies) using the plugin Variant Caller (VC) *v.*4.2-r88446. Hotspot and targeted regions, together with the json parameters files associated with the Ion AmpliSeq Colon and Lung Cancer Panel *v.*1 (Life Technologies), were then loaded into the VC plugin. All identified variants were visually confirmed by the Integrative Genomics viewer (IGV) [56]. To validate data, a second round of analysis was performed as follows: FastQ data, generated by the Ion PGM^TM^ semiconductor, were aligned to the Hg19 genome reference using BWA (Burrows-Wheeler Alignment)-MEM tool optimized for 70 bp–1 Mb long reads. Then, the resulting SAM (Sequence Alignment Map) files were converted and sorted to the BAM (binary version of SAM) format by SAMtools. Post-alignment steps included local realignment in the proximity of known indels and recalibration of the quality score, both performed by the Genome Analysis Toolkit (GATK) 2.5 Unified Genotyper (Realigner Target Creator, Indel Realigner and Base Recalibrator options) starting from sorted BAM files. Finally, GATK called variants in the target regions included in the BED file with Phred scores greater than 5 (those with a Phred score between 5 and 30 were marked as low-quality variants). Only Single Nucleotide Variants (SNVs) were considered, annotated by SNPEff, and compared to those called with the Ion Reporter™ tool [57]. All the samples belonging to the same patients were analyzed in multi-sample mode. Variants were annotated using the PolyPhen-2) [58], SIFT [59], and Genomic Evolutionary Rate Profiling (GERP) tools to predict the effect of missense mutations on the protein and calculate their conservation scores. Additionally, variants were named according to the Catalogue of Somatic Mutation in Cancer (COSMIC) [13] and Single Nucleotide Polymorphism Database (dbSNP). To distinguish common polymorphisms from rare variants, Global Minor Allele Frequency (GMAF) was calculated using the allele frequencies across all 1000 Genomes Phase I populations [60].

### 4.6. NGS Parameter Performance

To assess the performance parameters of our assay, two NSCLC cell lines and two DNA FFPE reference standards were sequenced according to our workflow. NGS data from the cell lines (NCI-H1650 and NCI-1975) were compared to genotypes obtained from published NGS data using Ion AmpliSeq™ Cancer Panel on Ion PGM sequencer (Life Technologies) [61]. Similarly, the data from the two FFPE reference standards were compared to genotype assessed by Horizon using droplet digital PCR. We also evaluated the performance parameters of our assay on WGA samples by comparing the mutation profiles of WGA amplified of NCI-H1650 scalar dilutions (10, 1, and 0.1 ng) with the corresponding unamplified cell line. The sensitivity, specificity, and PPV of the tests were calculated as described [62] and confidence intervals were calculated using the Confidence Interval Calculator (accessed on 1 February 2015).

### 4.7. Sanger Sequencing (SS) and Pyrosequencing (PS)

NGS variants with frequencies higher than 20% were validated by SS, using the specific gene primer of the panel as described by Coco (2012) [63]. Genetic variants with a frequency range from 5% to 20% were validated by PS. The primer sets for the PS assays (which include a PCR primer pair and a sequencing primer) were designed with the Pyrosequencing Assay Design software (Biotage, Uppsala, Sweden). All the primer sequences, the PCR amplification conditions, and the sequence to analyze are reported in Supplementary Table S5. The PCR reactions were performed by amplifying 100 ng of gDNA in a final volume of 50 μL containing 200 mol/L dNTPs, 1× Taq buffer, 1.5 mM MgCl_2_, 0.2 μM of each PCR primer, 1.5 U of AmpliTaq Gold^®^ DNA Polymerase (Life Technologies). The PCR program consists of 10 min at 95 °C and 45 cycles with 30 s at 95 °C, 30 s at specific annealing temperature of primer, and 30 s at 72 °C, followed by 5 min at 72 °C. The PS assays were performed with a PSQ 96MA instrument (Qiagen); the sequencing reactions were performed with the Pyro Gold reagent kit PSQ 96MA, according to the manufacturer’s instructions, and the sequencing analysis conducted with the PSQTM 96MA software *v*.2.02 (Biotage AB, Uppsala, Sweden).

## 5. Conclusions

In conclusion, here we demonstrate the feasibility of obtaining DNA suitable for NGS from FFPE tissues by performing an appropriate DNA quality control assay based on a multiplex PCR. Indeed, adequate quality control of extracted DNA represents the most critical step of the entire procedure and in particular gDNA from an FFPE biopsy, which is highly fragmented and limited. The assessment of the suitability of the starting DNA for subsequent sequencing is mandatory for reducing the time and costs of the downstream processes due to a possible sequencing failure.

In particular, our NGS-based workflow was successfully able to screen critical samples, such as FFPE tissues, demonstrating the possibility of transferring this novel technology into routine clinical context. Our criterion for considering a variant as true positive was a variant allele frequency greater than 5% with coverage higher than 500×.

We also report that VC is a reliable software program for identifying SNVs and indels using Ion PGM NGS data, that streamline and simplify the data analysis, annotation, and reporting of data, reducing the time to results. However, a second round of variant calling by GATK software is suggested, particularly in a starting setup. The inclusion of a reference standard, harboring mutation with AF at 5%, to sequence in each run as well as a validation step with an independent test to confirm clinically actionable mutations, are recommended to confidently call mutations in a clinical contest.

We also report a feasible sequencing workflow by starting from low-input gDNA to be applicable in the detection of ctDNA mutational profiles. However, to translate this technology into the disease monitoring and treatment of NSCLC, further validation studies on a greater number of samples, specifically on cfDNA from metastatic NSCLC patients, are required.

## Supplementary Materials

Supplementary materials can be found at http://www.mdpi.com/1422-0067/16/12/26129/s1.
